# Institutional Notes and News

**Published:** 1910-04-02

**Authors:** 


### Institutional Notes and News.

**St. Peter's Hospital for Stone, Covent Garden.**

The annual meeting of the Governors of this hospital was held in the board room of the Institution, Henrietta Street, Covent Garden, last week. Mr. F. A. Bevan, the Treasurer, who presided, in proposing the adoption of the report, said that this was the Jubilee year of the hospital. It was proposed to celebrate the occasion by a dinner, which was to be held on April 26, over which Lord Airedale had consented to preside. The committee were anxious to obtain a sum of £5,000 to clear off the deficit that existed and to provide funds for further equipment. The hospital had been a blessing to thousands of people, and he trusted that a liberal response would be made to the Jubilee appeal in order that the charity might continue its good work. Mr. Edwin Fox seconded the motion, and the report was adopted.

**New Works at Edinburgh Royal Infirmary.**

The reconstructed Out-patient Department opened last year has proved an unqualified success. Two work-rooms for the cutler have been added to the basement of the dispensary. A central instrument-room, long needed, and an office for the dispenser were constructed on the roof. A substantial addition to the storage accommodation has been obtained in rooms no longer needed for the medical out-patient department. The theatre of the professor of clinical surgery has been reconstructed and brought up to date, and will shortly be ready for occupation. In addition, a suite of rooms for various purposes has been provided behind the theatre; and a small emergency operating-room to the front, which can be rapidly heated, is to be at the disposal of any member of the staff requiring it. A small ward between Wards 10 and 12 has been converted into a clinical and operating theatre for the use of the new surgical charge, recently formed by the managers by taking a ward each from the two three-ward charges. This theatre came into use last January. A large tenement house in Archibald Place, with its back looking into the Infirmary grounds, has been purchased, and is now being converted into subsidiary quarters, with accommodation for 45 nurses and seven servant maids. It is expected to be ready for occupation before the spring. Accommodation has been provided for a new medical clinical laboratory, which is in course of preparation. The necessary furnishings, instruments, and apparatus will shortly be installed. The whole of the expense thereof and of the future maintenance has been defrayed by a generous and anonymous donor. The theatres in wards 35 and 36 have been re-arranged and modernised, and a large room in the basement of the fourth medical pavilion has been converted into a clinical class-room, thus doing away with the pressure and inconvenience of large classes of students in the wards.

**The Leicester Infirmary.**

In the unavoidable absence of the Duke of Rutland (President), through illness, the Mayor of Leicester (Councillor Geo. Chitham, J.P.) presided at the 137th anniversary meeting last week. There was a very large gathering of governors and subscribers. In moving the adoption of the report, Sir Edward Wood, the Chairman of Committees, referred to the great loss the Institution and the county had sustained by the death of the senior consulting surgeon (Sir Chas. Marriott, M.D., F.R.C.S.), who rendered invaluable service to the Institution as one of the hon. surgeons for upwards of forty years. He congratulated the governors on the finances. The expenditure for the year was ordinary £18,989 and extraordinary £3,976. Of the latter £2,756 was to repay the deficit that existed at the beginning of last year, which in turn had been reduced from £8,000, at which it stood at one time, £1,000 had been invested, £250 credited to contingencies, and £13 carried forward. The income was ordinary £18,989, extraordinary £3,946—total £22,935. The ordinary sources of income had been well maintained. Annual subscriptions had, however, only increased £120, in spite of the fact that upwards of £300 in new subscriptions had been brought in. This was one of the weaknesses of their finances. Their subscription list should certainly be £6,000 or £7,000, instead of under £4,000 per annum. The Institution had been saved by the contributions of the working men—whose contributions in pence and halfpence last year amounted to £13,000. Of that sum £8,837 was paid to the Infirmary, and £3,787 was devoted to the upkeep of the Saturday Hospital Society's convalescent work. One thousand and fifty-six men and women had been sent to convalescent homes by this fund. Church collections were a little above last year, but there was still room for improvement—£2,150 should be increased to £3,000. But they had been fortunate in having a good year in respect of legacies, which had relieved them of many difficulties; £3,946 had accrued from this source. They now had an enlarged and well-equipped Nurses' Home to maintain, and they would place 33 additional beds at the disposal of the public immediately, so that there was need of increasing the annual permanent income by £3,000. On the reconstruction scheme £100,000 had been expended, and the only sum now required to free them from debt was £557.

**The Wonder Year.**

Mr. G. Murray-Smith (the Vice-Chairman), in seconding, remarked that the past year was the wonder year of the Institution. The completion of the extension scheme had given the nurses a home which they fully deserved, and he thought the annual subscriptions should be, and would be in the near future, £6,000 per annum instead of £4,000. As to the need for larger income, he suggested that as a permanent monument to a dear relative nothing could be of more continual benefit than the endowment of a bed or a cot. The Mayor having also spoken, the report was enthusiastically adopted. Mr. S. F. Stone was re-elected treasurer on the motion of Mr. G. C. Franklin, F.R.C.S., seconded by Dr. R. Pratt. A vote of thanks to the hon. medical and surgical staff was moved by Mr. J. A. Corah, J.P., seconded by the Rev. A. I. Greaves, Mr. Cecil Marriott, F.R.C.S., replying. The retiring members of the board were re-elected on the motion of Sir Samuel Faire, seconded by Mr. G. Tempest Wade, J.P. Sir Arthur Hazlerigg, Bart., proposed the thanks of the meeting to Sir Edward Wood on the completion of his extension scheme, and pledging itself to support the appeal for funds to maintain the Institution in a state of efficiency. The extraordinary way in which Sir Edward Wood spotted where the money lay was really uncanny, as was the way in which he managed to get hold of it and take it to the Infirmary. He hesitated to compare Sir Edward Wood in that manner, but the Infirmary might be described as Sir Edward Wood's master—certainly he was its most valued servant. Mrs. Robert Martin, in seconding, alluded to the high standard of nursing which had always been set by Miss Rogers, and to the comfort which the new home was. The resolution was carried with applause.

**Worcester General Infirmary.**

The annual general meeting of governors was held in the board room on March 21, Mr. Francis J. A. Wood presiding. Unfortunately, few governors, others than those forming the committee, were present. There was some animated discussion on the report, which showed an excess of expenditure over ordinary income of £3,496 2s. 3d. As, however, legacies of under £1,000 are thrown into current account, and these had amounted to £2,227 7s. 10d., the deficit is not so great as at first sight appeared. It was suggested that patients able to do so should make some payment while in hospital. This was opposed on the grounds that it would introduce a condition foreign to the intention of a charitable institution. The need of a better children's ward, a better surgery, and an improved *x*-ray department were urgently brought to the notice of the governors, and an appeal to the public to provide these improvements suggested. On behalf of the honorary staff the expenditure was justified by the work done and the developments in the treatment of disease. It was pointed out that if the expenditure had doubled or trebled the benefits to the sick poor had quadrupled. The governors sanctioned the proposal "That the trustees be authorised to sell out of the funded property of the Infirmary, as may be required from time to time, sufficient to produce £3,000 to meet current expenses." The Chairman (Mr. Francis J. A. Wood) and the Vice-Chairman (the Rev. Canon Littleton Wheeler) were unanimously re-elected.

